# Adhésion médicamenteuse chez les coronariens âgés: expérience marocaine

**DOI:** 10.11604/pamj.2019.32.8.12415

**Published:** 2019-01-04

**Authors:** Imad Nouamou, Monia El Mourid, Yassine Ragbaoui, Rachida Habbal

**Affiliations:** 1 Service de Cardiologie, Centre Hospitalier Universitaire Ibn Rochd, Casablanca, Maroc

**Keywords:** Adhésion médicamenteuse, coronaropathie, sujet âgé, facteurs social et personnel, Medication adherence, coronary artery disease, elderly subject, social and personal factors

## Abstract

La prise en charge des coronaropathies a connu d'importants progrès. L’adhésion médicamenteuse chez les coronariens, en particulier chez les sujets âgés, demeure un défi majeur pour le contrôle de la maladie coronarienne et la prévention de ses complications. L’adhésion au traitement pourrait être influencé par la particularité démographique et socioéconomique précaire des pays africains. Nous avons réalisé une étude transversale de mars à octobre 2016 portant sur les patients âgés, suivis pour coronaropathie stable en ambulatoire. L'adhérence aux médicaments était évaluée par un questionnaire: Morisky Medication Adherence Scale. Les informations relatifs aux facteurs prédictifs d’adhésion médicamenteuse était dérivés d'un modèle d’adhésion multidimensionnel. Ont été inclus dans cette étude 115 patients âgés (âge > 65 ans). L'adhésion médicamenteuse était de 72,2% selon Morisky Medication Adherence Scale. La sédentarité a été retrouvée dans 59% des patients, l'hypertension artérielle et le diabète dans 42,6% et 41,7% des patients respectivement. Les facteurs prédictifs de mauvaise observance étaient: l'absence d'une mutuelle de santé (p = 0,02), la sévérité de la symptomatologie (p = 0,001), les patients ayant présenté un syndrome coronaire aigue (p = 0,006), le niveau de support social (p = 0,011) et la dépression (p = 0,006). L’adhésion médicamenteuse reste un problème de santé au Maroc notamment chez les personnes âgées. Les fournisseurs de soins de santé doivent être conscients des facteurs associés à une plus grande probabilité d'arrêt de traitement, en particulier les facteurs variables afin de mettre en place des stratégies adaptées pour améliorer l'observance du traitement.

## Abstract (en)

The treatment of coronary artery diseases has made significant progress. Medication adherence among patients with coronary artery disease, in particular among elderly patients, is a major challenge to disease control and prevention of its complications. Medication adherence could be influenced by the demographic and socio-economic vulnerable situation in the African countries. We conducted a cross-sectional study of elderly patients treated for stable coronary artery disease on an ambulatory basis from March to October 2016. Medication adherence was evaluated by a questionnaire: Morisky Medication Adherence Scale. The informations about predictive factors of medication adherence were obtained from a multidimensional adherence model. The study involved 115 elderly patients (age > 65 years). Medication adherence accounted for 72.2%, according to Morisky Medication Adherence Scale. Physical inactivity was found in 59% of patients, hypertension and diabetes in 42.6% and 41.7% of patients respectively. Poor compliance predictive factors were: the absence of a mutual health (p = 0.02), the severity of symptoms (p = 0.001), patients who had acute coronary syndrome (p = 0.006), the level of social support (p = 0.011) and depression (p = 0.006). Medication adherence is a health problem in Morocco, particularly among elderly subjects. Health care providers should be aware of factors associated with a higher probability of stopping treatment, in particular of variable factors, in order to implement personalized strategies to improve adherence to treatment.

## Introduction

L'adhésion du patient au traitement médicamenteux demeure un défi majeur pour le contrôle de la maladie coronarienne et la prévention de ses complications [1, 2]. Les patients âgés suivis pour une coronaropathie nécessitent un traitement par des médicaments multiples, ce qui les expose à un risque accru de non-adhésion aux médicaments. L'adhérence au traitement constitue un phénomène complexe influencé par un ensemble de facteurs élucidés par l’Organisation Mondiale de Santé avec un modèle d’adhésion multidimensionnel (MAM): facteurs liés au patient et à son état clinique, des facteurs liés au système des soins de santé ainsi que des facteurs socioéconomiques. Les interventions visant l’optimisation de l’adhésion du patient aux soins permettent de prévenir les hospitalisations et améliorent la morbi-mortalité cardiaque [3]. Ainsi, une connaissance de ces facteurs est nécessaire pour établir des interventions efficaces. Le but de cette étude était d'évaluer les facteurs liés à l'observance thérapeutique chez des patients coronariens âgés suivis en ambulatoire en utilisant le modèle d'adhérence multidimensionnelle de l'Organisation Mondiale de la Santé.

## Méthodes

Nous avons réalisé une étude transversale entre mars et octobre 2016 portant sur des patients coronariens en dehors d'un épisode aigu, âgés de plus de 65 ans suivis en consultation ambulatoire de Cardiologie du Centre Hospitalier Universitaire Ibn Rochd à Casablanca au Maroc. Nous avons inclus les patients dont le diagnostic de coronaropathie était confirmé par un cardiologue, ont été exclus de cette étude les patients souffrant de toute affection visuelle, cognitive ou psychiatrique importante qui empêcherait le participant de répondre au questionnaire. L’adhésion thérapeutique était mesurée par: Morisky Medication Adherence Scale (MMAS-8) qui contient huit questions. Le degré d'adhésion a été déterminé en fonction de la somme de toutes les réponses correctes: adhérence élevée (huit points), adhérence moyenne (6 à < 8 points) et mauvaise observance (< 6 points) [4]. Dans cette étude, les patients ont été considérés comme adhérents lorsqu'ils avaient un score égal à huit dans le MMAS-8.

Dans cette étude, on a recueilli les facteurs liés: 1) au patient: l'âge, le sexe, les connaissances vis-à-vis de la coronaropathie évaluées par le Coronary Artery Disease Education Questionnaire (CADE-Q) [5, 6]; 2) l’état clinique du patient: sévérité des symptômes, comorbidités évaluée par l'index de Charlson [7], dépression appréciée par le questionnaire «Patient Health Questionnaire» (PHQ9) [8]; 3) aux facteurs socioéconomiques: le niveau d'éducation, la présence d'une mutuelle de santé et le soutien social mesuré par l’échelle «Perceived Social Support Scale» [9].

Toutes les analyses de données ont été réalisées avec le SPSS version 21.0. L’analyse des données a commencé avec un examen descriptif de toutes les variables, le test de Khi2 était considéré comme significatif pour une valeur de p inférieure ou égale à 0,05.

**Considérations éthiques:** La participation à l'étude était volontaire. Le consentement était libre et éclairé, écrit ou verbal.

## Résultats

Nous avons colligé 115 patients âgés, suivis pour coronaropathie stable. La moyenne d’âge était de 71ans+/- 5,7 (65-90 ans). La majorité de nos patients étaient de sexe masculin avec un sexe ratio égal à 1,61, la sédentarité a été retrouvée chez 59% des patients, l'hypertension artérielle (HTA) et le diabète chez 42,6% et 41,7% des patients respectivement, la majorité de nos patients (70,4%) présentaient une comorbidité moyenne selon l'index de Charlson, 60% de nos patients ne présentaient pas de symptomatologie sévère: pas de limitation ou une limitation légère à l'activité physique (dyspnée = classe II NYHA ou Angor = classe II CCS), une fraction d'éjection du ventricule gauche (FEVG) entre 40 et 49% a été retrouvée dans 50,4% des patients. La consultation était motivée pour le suivi d'un syndrome coronarien aigu (SCA) chez 70 patients soit (60,9 %), contre 45 patients pour angor stable (39,1 %) (Tableau 1).

**Tableau 1:** Caractéristiques sociodémographique, clinique et psychosociale

	Effectif (n=115)	%
**Sexe**		
Masculin	71	61,7
Féminin	44	38,3
**Mutuelle de santé**	28	24,3
**Education**	29	25,2
**Connaissances**	32	27,8
**Facteurs de risque cardiovasculaire**		
HTA	39	42,6
Diabète	48	41,7
Dyslipidémie	29	25,2
Tabagisme	33	28,7
Sédentarité	68	59,1
**Comorbidités (Index de Charlson)**		
Faible	9	7,8
Moyenne	81	70,4
Elevée	21	18,3
Très élevée	2	1,7
Extrêmement élevée	2	1,7
**Symptomatologie**		
Dyspnée ou angor ≤ Stade II (NYHA^+^; CCS^++^)	69	60
Dyspnée ou angor ≥ Stade III (NYHA;CCS)	46	40
**FEVG**		
< 40%	15	13
40-49%	63	54,8
≥ 50%	37	32,2
**Tableau clinique**		
SCA	70	60,9
Sans SCA	45	39,1

+ NYHA: New York Heart Association

++ CCS: Canadian Cardiovascular Society

Le taux de prévalence d’adhérence médicamenteuse était de 72,2%. Les connaissances de nos patients concernant leurs pathologies étaient insuffisantes, seuls 32% des patients avaient un niveau de connaissance satisfaisant. Dans le groupe non adhérent: la dépression était retrouvée chez 21 patients, un niveau de soutien social faible chez 16 patients. Après une analyse multivariée, les facteurs prédictifs de mauvaise observance étaient: l'absence d'une mutuelle de santé (p = 0,02), une symptomatologie non sévère (p = 0,001), les patients ayant présenté un SCA (p = 0,006), le niveau de support social (p = 0,011) et la dépression (p = 0,006) (Tableau 2).

**Tableau 2:** Facteurs prédictifs de l'adhésion médicamenteuse

	Observant n (%)	Non observant n (%)	P
**Sexe**			
Masculin	50	21	0,59
Féminin	33	11	
**Mutuelle de santé**	25	3	0,02^+^
**Education**	18	11	0,16
**Connaissances**	24	8	0,675
**Facteurs de risque cardiovasculaire**			
HTA	35	14	0,878
Diabète	33	15	0,488
Dyslipidémie	21	8	0,973
Tabagisme	22	11	0,403
Sédentarité	49	19	0,974
**Symptomatologie**			
Dyspnée ou angor ≤ Stade II (NYHA;CCS)	42	27	0,001^+^
Dyspnée ou angor ≥ Stade III (NYHA;CCS)	41	5	
**Clinique**			0,006^+^
SCA	57	13	
Sans SCA	26	19	
**Soutien social**	62	16	0,011^+^
**Dépression**	31	21	0,006^+^

+ NYHA: New York Heart Association

++ CCS: Canadian Cardiovascular Society

## Discussion

Peu d'études épidémiologiques sur l'adhérence médicamenteuse en cas de coronaropathie existent. A notre connaissance c'est la première étude qui porte sur les coronariens âgés. En effet la littérature n'offre que peu de recherches directement comparables à celles de notre étude. Comme la population âgée est en croissance rapide dans le monde entier [10], au Maroc les personnes âgées représenteraient 15,4% de la population totale à l'horizon 2030, soit le double du niveau actuel [11]. Par conséquent, il nous a semblé nécessaire d'analyser la particularité de cette catégorie fragile de nos patients.

L’adhésion thérapeutique est définie par le degré de respect ou d'écart entre les prescriptions et les pratiques du patient en termes de santé. Son évaluation et sa promotion permettent d’améliorer le pronostic des patients [12]. La difficulté de l’évaluation de l'adhérence médicamenteuse réside dans la disparité et la diversité des critères d’appréciation [13, 14]. Les méthodes de mesure de l'adhérence peuvent être classées en méthode directe et indirecte. Les méthodes directes comprennent la mesure de la concentration de médicament dans le sang et la mesure du marqueur biologique dans le corps. Les méthodes indirectes comprennent les questionnaires, l’auto-évaluation, le décompte des comprimés restants, la fréquence de renouvellement des ordonnances et les piluliers électroniques. L'évaluation par un questionnaire est l'une des méthodes indirectes importantes pour mesurer l'adhésion aux médicaments. C'est une méthode simple efficace et couramment utilisée dans le cadre clinique [15, 16].

Dans notre étude le taux de prévalence était de 72,2%, ce qui rejoint les données de la littérature qui varient dans de larges proportions 21% à 74% selon les études [17, 18]. Dans notre population nous n'avons pas trouvé de différence significative entre les deux sexes. Dans la littérature, les résultats des études divergent. Certaines études rejoignent nos données [19, 20], d'autres montrent que le sexe est un facteur influençant l'adhérence médicamenteuse [14, 21]. Le niveau d'éducation et de connaissance concernant la maladie coronarienne chez nos patients n'était pas associé à une mauvaise adhérence médicamenteuse p = 0,16 et p = 0,67 respectivement alors que d'autres études ont montré qu'un niveau bas d'éducation et de connaissance était associé à une mauvais observance [18, 22, 23].

Plusieurs études ont montré le rôle du coût du traitement sur l'adhérence au traitement. Nos patients qui n'avaient pas de mutuelle de santé étaient moins adhérents aux traitements (p = 0,02), le coût élevé du traitement étant pris en charge par le patient, ceci peut expliquer l'effet défavorable du bas niveau socioéconomique sur la mauvaise observance thérapeutique en particulier dans un pays comme le Maroc [22, 24, 25]. Les facteurs psychosociaux tels que le support social et la dépression étaient des facteurs prédictifs d'une mauvaise observance: les patients qui ont reçu un soutien social par des membres de leur famille ou de leurs amis étaient plus adhérents (p = 0,01). Dans une étude observationnelle, le support social prévoyait une augmentation de plus de deux fois l'adhésion aux médicaments chez les patients qui avaient deux sources de soutien ou plus après un an [26]. Notre travail appuie les études qui ont identifié la dépression comme un puissant prédicteur de la non-adhésion des médicaments chez les coronariens âgés [19, 27].

D'autres facteurs influençaient l'adhésion thérapeutique: les patients qui n'ont pas présenté un syndrome coronaire aigu et les patients avec une symptomatologie non sévère étaient moins adhérents aux traitements avec p égal à 0,006 et 0,001 respectivement, la relation entre l'adhérence aux médicaments et la classe fonctionnelle était rarement étudiée [28, 29], une étude multivariée a montré que, plus la gêne fonctionnelle est importante plus l'observance aux traitements est difficile, contrairement à notre étude [30].

Nous proposons quelques interventions qui ont démontré un certain succès comme la réduction du nombre de doses quotidiennes de médicaments aux maximum, la motivation des patients en leurs expliquant les risques et bénéfices de l'adhérence aux traitements, ainsi que la prise en charge des facteurs psychosociaux tels que la dépression et le soutien social pour améliorer l'observance des médicaments chez les patients atteints d'une maladie coronarienne en particulier les sujets âgés.

Notre étude présente quelques limites: l'absence de consensus et du "gold standards" concernant la meilleure méthode pour la mesure de l'adhérence ainsi que la taille de notre échantillon. Les résultats de cette étude doivent être confirmés dans une plus grande population.

## Conclusion

L’adhésion médicamenteuse reste un problème de santé au Maroc notamment chez les personnes âgées. Notre étude montre que l'adhésion médicamenteuse reste insuffisante. Les fournisseurs de soins de santé doivent être conscients des facteurs prédictifs d'une mauvaise adhérence aux traitements en particulier les facteurs variables afin de mettre en place des stratégies efficaces et non onéreuse pour améliorer l'observance du traitement chez cette population.

### Etat des connaissances actuelles sur le sujet

- Facteurs d’adhésion médicamenteuse dans les pays développés;
- Influence de l'adhésion médicamenteuse sur le pronostic;
- Rôle des facteurs psychosociaux dans l'adhérence médicamenteuse.

### Contribution de notre étude à la connaissance

- La première étude au Maroc qui traite de l’adhésion médicamenteuse chez les patients avec coronaropathie;
- Particularité des patients âgés;
- Rôle de la sévérité de la symptomatologie et du tableau clinique dans l'adhérence médicamenteuse.

## Conflits d’intérêts

Les auteurs ne déclarent aucun conflit d’intérêts.

## References

[1] American Heart Association (2012). Heart disease and stroke statistics - 2012 Update: a report from the American Heart Association. Circulation.

[2] Ho PM, Magid DJ, Shetterly SM, Olson KL, Maddox TM, Peterson PN, Masoudi FA, Rumsfeld JS (2008). Medication non adherence is associated with a broad range of adverse outcomes in patients with coronary artery disease. Am Heart J.

[3] Rich MW, Beckham V, Wittenberg C, Leven CL, Freedland KE, Carney RM (1995). A multidisciplinary intervention to prevent the readmission of elderly patients with congestive heart failure. N Engl J Med.

[4] Tan X, Patel I, Chang J (2014). Review of the four item Morisky Medication Adherence Scale (MMAS-4) and eight item Morisky Medication Adherence Scale (MMAS-8). Innovations in Pharmacy.

[5] Ghisi GL, Durieux A, Manfroi WC, Herdy AH, Carvalho T, Andrade A (2010). Construction and validation of the CADE-Q for patient education in cardiac rehabilitation programs. Arq Bras Cardiol.

[6] De Melo Ghisi GL, Oh P, Thomas S, Benetti M (2013). Development and validation of an English version of the Coronary Artery Disease Education Questionnaire (CADE-Q). Eur J Prev Cardiol.

[7] Charlson ME, Pompei P, Ales KL, MacKenzie CR (1987). A new method of classifying prognostic comorbidity in longitudinal studies: development and validation. J Chronic Dis.

[8] Kroenke K, Spitzer RL, Williams JB (2001). The PHQ-9: validity of a brief depression severity measure. J Gen Intern Med.

[9] Canty Mitchell J, Zimet GD (2000). Psychometric properties of the multidimensional scale of perceived social support in urban adolescents. Am J Community Psychol.

[10] United Nations World population aging: 1950–2050.

[11] Haut Commissariat au Plan Analyse des résultats de l’Enquête nationale sur les personnes âgées ENPA 2006.

[12] Ferrières J, Durack-Bown I, Giral P, Chaderevian R, Benkritly A, Bruckert E (2006). Patient education and patient at risk: a new approach in cardiology. Ann Cardiol Angeiol.

[13] Ho PM, Bryson CL, Rumsfeld JS (2009). Medication adherence, its importance in cardiovascular outcomes. Circulation.

[14] Gehi AK, Ali S, Na B, Whooley MA (2007). Self-reported medication adherence and cardiovascular events in patients with stable coronary heart disease: the heart and soul study. Arch Intern Med.

[15] Wai Yin Lam, Paula Fresco (2015). Medication adherence measures: an overview. Biomed Res Int.

[16] Walsh JC, Mandalia S, Gazzard BG (2002). Responses to a 1 month self-report on adherence to antiretroviral therapy are consistent with electronic data and virological treatment outcome. Aids.

[17] Scheen AJ (1999). La non-observance thérapeutique: problème majeur pour la prévention des maladies cardiovasculaires. Rev Med Liege.

[18] Kulkarni SP, Alexander KP, Lytle B, Heiss G, Peterson ED (2006). Longterm adherence with cardiovascular drug regimens. Am Heart J.

[19] Park LG, Howie-Esquivel J, Whooley MA, Dracup K (2015). Psychosocial factors and medication adherence among patients with coronary heart disease: a text messaging intervention. Eur J Cardiovasc Nurs.

[20] Molloy GJ, Messerli-Bürgy N, Hutton G, Wikman A, Perkins-Porras L, Steptoe A (2014). Intentional and unintentional non-adherence to medications following an acute coronary syndrome: A longitudinal study. J Psychosom Res.

[21] Ivers NM, Schwalm JD, Jackevicius CA, Guo H, Tu JV, Natarajan M (2013). Length of initial prescription at hospital discharge and long term medication adherence for elderly patients with coronary artery disease: a population level study. Can J Cardiol.

[22] Jackevicius CA, Li P, Tu JV (2008). Prevalence, predictors and outcomes of primary non adherence after acute myocardial infarction. Circulation.

[23] Newby LK, Lapointe NMA, Chen AY, Kramer JM, Hammill BG, Delong RE (2006). Long-term adherence to evidence-based secondary prevention therapies in coronary artery disease. Circulation.

[24] Cournot M, Cambou JP, Quentzel S (2005). Motifs de sous-utilisation des thérapeutiques chez les coronariens de plus de 70 ans. Ann Cardiol Angeiol (Paris).

[25] Roth GA, Morden NE, Zhou W, Malenka DJ, Skinner J (2012). Clopidogrel use and early outcomes among older patients receiving a drug eluting coronary artery stent. Circ Cardiovasc Qual Outcomes.

[26] Molloy GJ, Perkins-Porras L, Strike PC (2008). Social networks and partner stress as predictors of adherence to medication, rehabilitation attendance and quality of life following acute coronary syndrome. Health Psychol.

[27] Dempe C, Junger J, Hoppe S (2013). Association of anxious and depressive symptoms with medication nonadherence in patients with stable coronary artery disease. J Psychosom Res.

[28] Rodgers PT, Ruffin DM (1998). Medication nonadherence: Part II- A pilot study in patients with congestive heart failure. Manag Care Interface.

[29] Roe CM, Motheral BR, Teitelbaum F, Rich MW (1999). Angiotensin-converting enzyme inhibitor compliance and dosing among patients with heart failure.

[30] Wu JR, Moser DK, Chung ML, Lennie TA (2008). Predictors of medication adherence using a multidimensional adherence model in patients with heart Failure. J Card Fail.

